# Primal-dual for classification with rejection (PD-CR): a novel method for classification and feature selection—an application in metabolomics studies

**DOI:** 10.1186/s12859-021-04478-w

**Published:** 2021-12-15

**Authors:** David Chardin, Olivier Humbert, Caroline Bailleux, Fanny Burel-Vandenbos, Valerie Rigau, Thierry Pourcher, Michel Barlaud

**Affiliations:** 1 Transporters in imaging and Radiotherapy in Oncology (TIRO), Direction de la Recherche Fondamentale (DRF), Institute des sciences du vivant Fréderic Joliot, Commissariat à l’Energie Atomique et aux énergies alternatives (CEA), Université Côte d’Azur (UCA), Nice, France; 2 Department of Nuclear Medicine, Centre Antoine Lacassagne, Université Côte d’Azur (UCA), Nice, France; 3 Laboratoire d’Informatique, Signaux et Systèmes de Sophia Antipolis (I3S), Université Côte d’Azur (UCA), Centre de Recherche Scientifique (CNRS), Sophia Antipolis, France; 4 Department of Oncology, Centre Antoine Lacassagne, Université Côte d’Azur (UCA), Nice, France; 5 Central Laboratory of Pathology, University Hospital and Institute of Biology Valrose, Inserm U1091 – CNRS UMR7277, University Côte d’Azur, Nice, France; 6 Department of Pathology and Oncobiology, University Hospital, Montpellier, France; 7 Institute for Neurosciences of Montpellier, INSERM U1051, Montpellier, France

## Abstract

**Background:**

Supervised classification methods have been used for many years for feature selection in metabolomics and other omics studies. We developed a novel primal-dual based classification method (PD-CR) that can perform classification with rejection and feature selection on high dimensional datasets. PD-CR projects data onto a low dimension space and performs classification by minimizing an appropriate quadratic cost. It simultaneously optimizes the selected features and the prediction accuracy with a new tailored, constrained primal-dual method. The primal-dual framework is general enough to encompass various robust losses and to allow for convergence analysis. Here, we compare PD-CR to three commonly used methods: partial least squares discriminant analysis (PLS-DA), random forests and support vector machines (SVM). We analyzed two metabolomics datasets: one urinary metabolomics dataset concerning lung cancer patients and healthy controls; and a metabolomics dataset obtained from frozen glial tumor samples with mutated isocitrate dehydrogenase (IDH) or wild-type IDH.

**Results:**

PD-CR was more accurate than PLS-DA, Random Forests and SVM for classification using the 2 metabolomics datasets. It also selected biologically relevant metabolites. PD-CR has the advantage of providing a confidence score for each prediction, which can be used to perform classification with rejection. This substantially reduces the False Discovery Rate.

**Conclusion:**

PD-CR is an accurate method for classification of metabolomics datasets which can outperform PLS-DA, Random Forests and SVM while selecting biologically relevant features. Furthermore the confidence score provided with PD-CR can be used to perform classification with rejection and reduce the false discovery rate.

**Supplementary Information:**

The online version contains supplementary material available at 10.1186/s12859-021-04478-w.

## Introduction

Among the different omics fields, metabolomics is the most recent and provides new insights for a global study of biological systems. Metabolomics is a rapidly growing and promising field of research in biology and healthcare. Metabolomics approaches are based on the determination of the levels of different small molecules or metabolites in biological samples (tissue, cells, serum, urine...). Interestingly, ever since the early metabolomics studies, supervised classification methods have been used for the analysis of the related datasets. One of the initial aims of metabolomic studies was to establish useful biomarkers, indicative of specific physiological states or aberrations. The challenge now is to understand the mechanisms by which changes in the metabolome are implicated in different phenotypic outcomes in a complex systems biology approach [1, 2].

Most metabolomics studies generate complex multivariate datasets including varying correlations between features and systematic noise. Therefore, multivariate data analysis methods are needed to explore these datasets. One of the most frequently used methods for metabolomics analyses is Partial Least Squares-Discriminant Analysis (PLS-DA) [3, 4].

PLS-DA is a chemometric technique used to optimize separation between different classes of samples, which is accomplished by linking two data matrices: X (raw metabolomic data) and Y (class membership). It has the advantage of handling highly collinear and noisy data. Yet, it has some drawbacks and needs to be handled with caution. Indeed it has been reported that PLS-DA can: 1. Lead to over-fitting when the number of variables significantly exceeds the number of samples. Indeed, in this setting, the model is likely to lead to accurate classification by chance, based on irrelevant features [5]; 2. Have difficulties when few variables are responsible for the separation between two or more classes and, therefore, require a larger number of variables to achieve a good prediction accuracy [6]; and finally, 3. Lead to an over-optimistic understanding of the separation between two or more classes [7].

Continuous effort is being made to provide new statistical tools to tackle these drawbacks [8]. Some authors use Random Forests [9] as an alternative to PLS-DA for metabolomics studies [10]. Random Forests are based on the bagging algorithm and use an Ensemble Learning technique. Random Forests create a large number of decision trees and combine their outputs. Yet, Random Forests have significant drawbacks. For instance, they tend to over-fit when using noisy datasets. Furthermore, the main disadvantage of Random Forests is their complexity. Indeed, they are much harder and time-consuming to construct, require more computational resources and are less intuitive than decision trees. Furthermore this complexity significantly hampers their interpretability. Support Vector Machines (SVM) are another option [11, 12] but have similar drawbacks as Random Forests and are particularly consuming in computational resources.

Mathematics I3S partner has recently introduced a new tailored, constrained primal-dual method for supervised classification and feature selection [13]. This method has the significant advantage of providing a trustworthy confidence index with each prediction, which we use to define a new classifier with rejection. This is particularly useful in the context of clinical decision making as it diminishes the number of false positive and false negative results. Moreover, we believe this method out-performs other methods in terms of accuracy and feature selection.

Although there are many machine learning methods for feature selection such as LASSO [14, 15], Discriminant analysis [16], Proximal methods [17, 18] and Boosting [19, 20], here we compare our novel Primal-Dual method for Classification with Rejection (PD-CR) to the state of the art PLS-DA and Random Forests and SVM classification methods frequently used in metabolomics studies.

## Methods

### Mathematical background

#### Robust classification and regression using $\ell _1$ centers

Mathematically, classification problems can be described as follows:

Let *X* be the $m \times d$ data matrix made of *m* line samples $x_1,\dots ,x_m$ that belong to the *d*-dimensional space of features.

Let $Y \in \{0,1\}^{m\times k}$ be the matrix of labels where $k \ge 2$ is the number of clusters. Each line of *Y* has exactly one nonzero element equal to one, $y_{ij}=1$ indicating that the sample $x_i$ belongs to the *j*-th cluster. Projecting the data in lower dimension is crucial to be able to separate them accurately.

Let *W* be the $d \times k$ projection matrix, where $k \ll d$. (Note that the dimension of the projection space is equal to the number of clusters.)

The goal of the supervised classification method is to find the best possible values for the projection matrix *W*.

Sparse learning based methods have received a lot of attention in the last decade because of their high level of performance. The basic idea is to use a sparse regularizer that forces some coefficients to be zero. To achieve feature selection, the *Least Absolute Shrinkage and Selection Operator* (LASSO) formulation [14, 21–25] adds an $\ell _1$ penalty term to the classification cost. An accurate criterion is based on the sum of the square difference (used in k-means [26]) and can be cast as follows:

1 $$\begin{aligned} \Vert Y\mu -XW\Vert _F^2 = \sum _{j=1}^{k} \sum _{l \in C_j} \Vert (XW)(l,:)-\mu _j \Vert _2^2, \end{aligned}$$

where $C_j \subset \{1,\dots ,m\}$ denotes the *j*-th class, and where the row vector $\mu _j$ is the centroid of this class. Therefore, the matrix of centers $\mu$ is a square matrix of order *k*. It is well known that the Frobenius norm is sensitive to outliers. To address this, we have improved the approach by replacing the Frobenius norm by the $\ell _1$ norm of the loss term as follows:

2 $$\begin{aligned} \Vert Y\mu -XW\Vert _1=\sum _{j=1}^{k} \sum _{l \in C_j} \Vert (XW)(l,:)-\mu _j \Vert _1. \end{aligned}$$

where $C_j \subset \{1,\dots ,m\}$ denotes the *j*-th cluster, and where $\mu _j:=\mu (j,:)$ is the *j*-th line of $\mu$. In our method, we simultaneously optimize $(W,\mu )$, adding some *ad hoc* penalty to break homogeneity and avoid the trivial solution $(W,\mu )=(0,0)$.

Using both the projection *W* and the centers $\mu$ learnt during the training step, a new query *x* in the test set (a dimension *d* row vector) is classified according to the following rule: it belongs to the cluster number $j^*$ if and only if

3 $$\begin{aligned} j^* \in \arg \min _{j=1,\dots ,k} \Vert \mu _j-xW\Vert _1. \end{aligned}$$

#### Primal-dual scheme, constrained formulation

To handle features with a high correlation, we consider a convex constrained supervised classification problem. However the drawback of the term $\Vert Y \mu -XW\Vert _1$ is that it enforces equality of the two matrices out of a sparse set: hence it tunes the parameters to enforce a perfect matching of the training data. We replace the 1-norm with the robust “Huber function” [13]. If $h_\delta (t) = t^2/(2\delta )$ for $|t|\le \delta$ and $|t|-\delta /2$ for $|t|\ge \delta$.

We obtain the following criterion

4 $$\begin{aligned} \min _{(W,\mu )} h_\delta (Y \mu -XW) + \frac{\rho }{2}\Vert I_k-\mu \Vert _F^2 \text { s.t. } \quad \Vert W\Vert _1 \le \eta . \end{aligned}$$

We can tune a primal-dual method to solve this problem with Algorithm 1 (See [13] and [27] for details) 
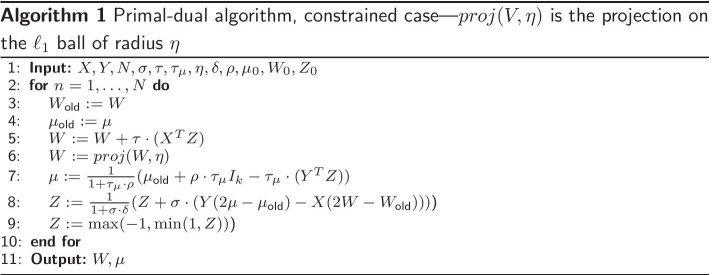


#### Classification with rejection using a confidence Score for the Prediction (CSP)

False positive (FP) and false negative (FN) results are an important issue for diagnostic tools in medicine. One way to diminish the number of FP and FN results is to use classification with rejection [19, 28] for which classifiers are allowed to report “I don’t know”. This type of classification enables the incorporation of doubt in the results if the observation x is too hard to classify. Here, we propose to use a confidence score for the prediction (CSP) to devise a classifier with rejection.

In our analysis we only had two clusters with centers $\mu _1$ and $\mu _2$ Lets recall that the predicted label $j^*$ of a sample x is given by

5 $$\begin{aligned} j^* \in \arg \min _{j=1,\dots ,2} \Vert \mu _j-xW\Vert _1. \end{aligned}$$

We can compute the distances of sample x to the two centroids, respectively. $d_1=\Vert \mu _1-x W\Vert _1$ and $d_2=\Vert \mu _2-x W\Vert _1$ and we propose a confidence indicator for sample x as follows:

6 $$\begin{aligned} \rho (x) = \frac{d_1-d_2}{d_1+d_2} \end{aligned}$$

Thus, the CSP $\rho (x)$ is a value ranging from -1 to 1. The closer the CSP $\rho (x)$ is to +1 or -1 depending on the predicted class, the higher the confidence for the prediction will be.

Thus if $\epsilon$ is a given threshold parameter, we can perform classification with rejection by rejecting binary classification for samples with an absolute value of CSP $\rho (x)$ under this threshold. The labels will then be predicted as follows:

7 $$\begin{aligned} Label = {\left\{ \begin{array}{ll} -1 &{}\text{ if } \rho (x)< -\epsilon \\ 0 &{}\text{ if } - \epsilon< \rho (x)< \epsilon \\ 1 &{}\text{ if } \rho (x)> \epsilon \end{array}\right. } \end{aligned}$$

We can then study the False Discovery Rate (FDR) $FDR= FP+ FN$ as a function of parameter $\epsilon$.

## Comparison to PLS-DA, Random Forests and SVM using 2 datasets

To compare PD-CR to the standard PLS-DA, Random Forests and SVM classification methods in terms of accuracy and feature selection, we tested the four methods on two metabolomic datasets named “BRAIN” and “LUNG”. Accuracies and feature selection for each method were obtained using 4 fold-cross validation with varying random seeds. We also provide the results with a a new version of PD-CR minimizing the $\ell 2$ norm PD-CR $\ell 2$ (See Algorithm 6 *https* : //*arxiv*.*org*/*pdf*/1902.01600.*pdf*).

### LUNG dataset

The LUNG dataset was provided by Mathe et al. This dataset includes metabolomics data concerning urine samples from 469 Non-Small Cell Lung Cancer (NSCLC) patients prior to treatment and 536 controls collected from 1998 to 2007 in seven hospitals and in the Department of Motor Vehicles (DMV) from the greater Baltimore, Maryland area. Urine samples were analyzed using an unbiased metabolomics LC-MS/MS approach. This dataset is available from the MetaboLights database (study identifier MTBLS28)

Mathe et al. used Random Forests to classify patients as lung cancer patients or controls [10]. The aim was to create a new screening test for lung cancer, based on metabolomics data from urine. Lung cancer is one of the most common cancers and it is well established that early diagnosis is essential for treatment. An efficient screening method based on urinary metabolomics would be of great benefit.

### BRAIN dataset

The BRAIN dataset was obtained from a metabolomic study performed by our biological team (TIRO) on frozen samples of glial tumors. The samples were provided by the university hospitals of Nice and Montpellier (France). Metabolite extracts were prepared and analyzed in the TIRO laboratory (Nice, France). With this dataset, the goal was to create a model that accurately discriminated between mutated isocitrate dehydrogenase (IDH) and IDH wild-type glial tumors. This mutation is a key component of the World Health Organization classification of glial tumors [29]. The mutational status is usually assessed by IDH1 (R132H)-specific (H09) immunohistochemistry. Yet this technique can lead to False-Negative results [30], which can only be identified by sequencing. An accurate metabolomic based test, able to assess the IDH mutational status, could be a promising solution to this problem.

These samples were retrospectively collected from two declared biobanks from the Central Pathology Laboratory of the Hospital of Nice and from the Center of Biological Resources of Montpellier (Plateforme CRB-CHUM). Consent or non-opposition was verified for every participant. For every participant, the IDH mutational status was assessed using immunohistochemistry and pyrosequencing for immunonegative cases.

Samples of brain tumors were analyzed using Liquid Chromatography coupled to tandem Mass Spectrometry (LC-MS/MS) in an unbiased metabolomics approach, as performed in a previous metabolomics study [xxx].

The details of the analysis are available in Additional file 1.

### Data Filtering and Pre-processing

Our laboratory performed the LC-MS/MS analysis for the BRAIN dataset. Therefore, we could apply different levels of filtering on this dataset. After processing of the raw data using MZmine 2.39 software, two types of filtering were applied to the BRAIN dataset, minimal and maximal filtering. The minimal filtering only removed metabolites for which a spike was detected in less than 10 percent of the samples. The maximal filtering removed all unidentified metabolites as well as metabolites that did not have an isotopic pattern. This filtering method is frequently used for metabolomic studies and diminishes the number of noisy features in the dataset. Furthermore, it diminishes the time necessary for data processing because it diminishes the data volume. Unfortunately, any filtering will necessarily come with a high risk of removing some relevant features which is also the case with this filtering method. Using the two BRAIN datasets, we aimed to assess how the filtering affected the results of the different classification methods. The LUNG dataset was used as it was published, without additional normalization or filtering.

### Comparison to other methods

Before comparison, the data were pre-processed as follows:

(i) Log-transformation for the following benefits: Reducing heteroscedasticity and thus the bias on regression and transforming multiplicative noise into additive noise,
(ii) Mean centering and scaling [31].

PD-CR [13] was compared to PLS-DA [32], Random Forests (with 100 and 400 trees) [9] and SVM using the sklearn python package.

Additionally, we evaluated the impact of the use of the Huber loss in PD-CR compared to the use of the $\ell 2$ loss.

Parameters $\sigma ,\tau ,\delta$ and $\rho$ were set according to results obtained using various datasets in an initial step [13] and were not further tuned. Parameter $\eta$, which affects the feature selection step was manually tuned to fit the number of features in the datasets and to maximize accuracy after cross validation.

We computed the accuracy of the 4 classification methods for the two metabolomics datasets using 4-fold cross-validation (Script “PD-CR vs PLS-DA, RF and SVM” on https://github.com/tirolab/PD-CR). The selected metabolites were analyzed and compared between methods for the metabolomics datasets.

For PD-CR, we plotted the histograms of the CSP $\rho (x)$ and the probability distribution function (PDF) as well as the False Discovery Rate (FDR =(FP+FN)/total) and the rate of rejected samples (RRS = rejected samples/total samples) depending on epsilon (the CSP threshold) (Script “rhoComputing” on https://github.com/tirolab/PD-CR).

## Results

The characteristics of the two metabolomics datasets are presented in Table 1.

The LUNG dataset included a large number of patients (a little over 1,000) with an equivalent number of features (a little under 3,000) and the BRAIN dataset included a smaller number of patients (88) with a much higher number of features. While obtaining metabolomics data concerning as many patients as there are in the LUNG dataset is remarkable, the number of patients in the BRAIN dataset is closer to the number of patients in most metabolomics studies.

**Table 1:** Overview of the datasets

Dataset	No. of samples	No. of features	Sample type
LUNG	1005	2944	Urine
BRAIN	88	25,286	Glial tumor tissue

### LUNG

**Table 2:** LUNG dataset: mean accuracy using 3 seeds and 4-fold cross validation: comparison with PLS-DA, Random forest and Best SVM

LUNG	PD-CR	PD-CR $\ell _2$	PLS-DA	RF (100 trees)	RF (400 trees)	SVM
Accuracy $\%$	79.44	78.3	76.56	71.31	72.44	76.25
AUC	79.97	${-}$	74.05	73.38	74.50	76.64
Time (s)	0.11	0.11	0.09	0.89	3.47	85.6

As shown in Table 2, PD-CR outperformed PD-CR $\ell _2$, PLS-DA, Random Forests (400 trees) and SVM by $1.1 \%$, $2.8 \%$, $7 \%$ and $3.1 \%$ respectively.

**Fig. 1:**
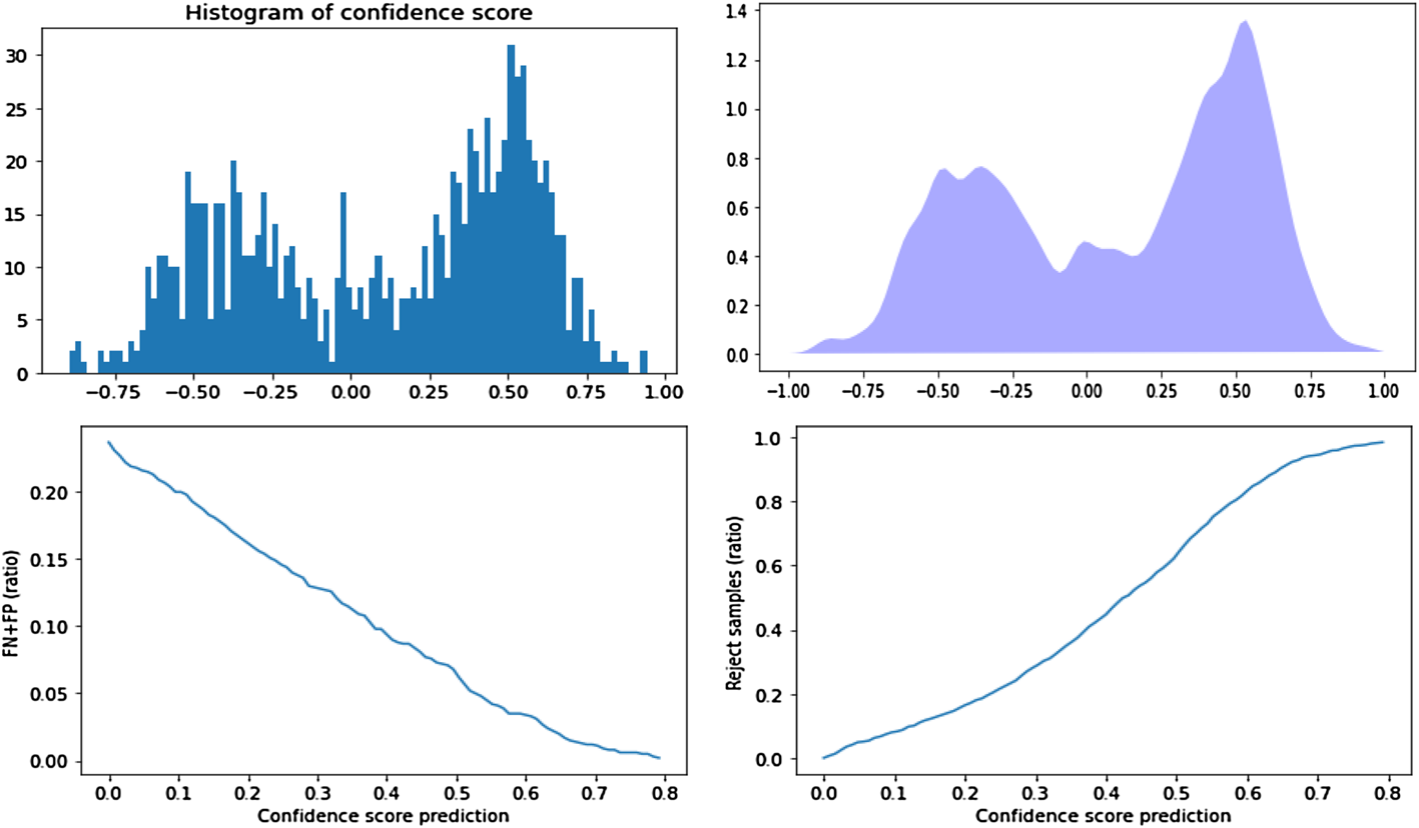
Distribution of the confidence score for the prediction (CSP) on the Lung dataset and impact of using CSP for classification with rejection on the false discovery rate (FDR). From Left to right and top to bottom: Histogram of the CSP, Kernel density estimation; FDR as a function of CSP after classification with rejection, rate of rejected samples as a function of CSP after classification with rejection. As expected for a pertinent confidence score, the FDR diminishes when using a higher CSP threshold for classification with rejection

Even though an accuracy of 79.44% may be high enough to consider using our PD-CR method and urinary metabolomics for the screening of lung cancer, Fig. 1 shows that the accuracy may be even higher if the CSP is taken into account and if it is used to perform classification with rejection. Indeed, in Fig. 1 the top left shows the histogram of the CSP and the top right the kernel probability distribution function (PDF). We can see that healthy controls and cancer patients are predicted with an equally high confidence. On the bottom left the False Discovery Rate ($FDR =(FP+FN)/total\ samples$) decreases as the confidence score threshold increases, but as shown in the bottom right, the rate of rejected samples ($RRS = rejected\ samples/total\ samples$) increases.

**Table 3:** Top 10 features selected by random forests, PLS-DA, PD-CR and SVM in the LUNG dataset

RF	PLS-DA	PD-CR	SVM
MZ 264.1215224	MZ 264.1215224	MZ 264.1215224	MZ 264.1215224
MZ 656.2017529	MZ 126.9069343	MZ 308.0984878	MZ 308.0984878
MZ 441.1613664	MZ 170.0605916	MZ 126.9069343	MZ 247.0970455
MZ 584.2670695	MZ 613.3595637	MZ 613.3595637	MZ 613.3595637
MZ 247.0970455	MZ 243.1004849	MZ 243.1004849	MZ 615.0353192
MZ 486.2571336	MZ 486.2571336	MZ 247.0970455	MZ 372.9232556
MZ 308.0984878	MZ 308.0984878	MZ 332.0963401	MZ 441.1613664
MZ 204.1345526	MZ 561.3432022	MZ 441.1613664	MZ 370.0525988
MZ 247.1384435	MZ 94.06574518	MZ 94.06574518	MZ 423.0084949
MZ 447.10803	MZ 269.1280232	MZ 561.3432022	MZ 332.0963401

As shown in Table 3, PD-CR selected “MZ 264.1215224” for a molecular ion at m/z 264.1215224 and “MZ 308.0984878” for a molecular ion at m/z 308.0984878 as the top two features.

**Fig. 2:**
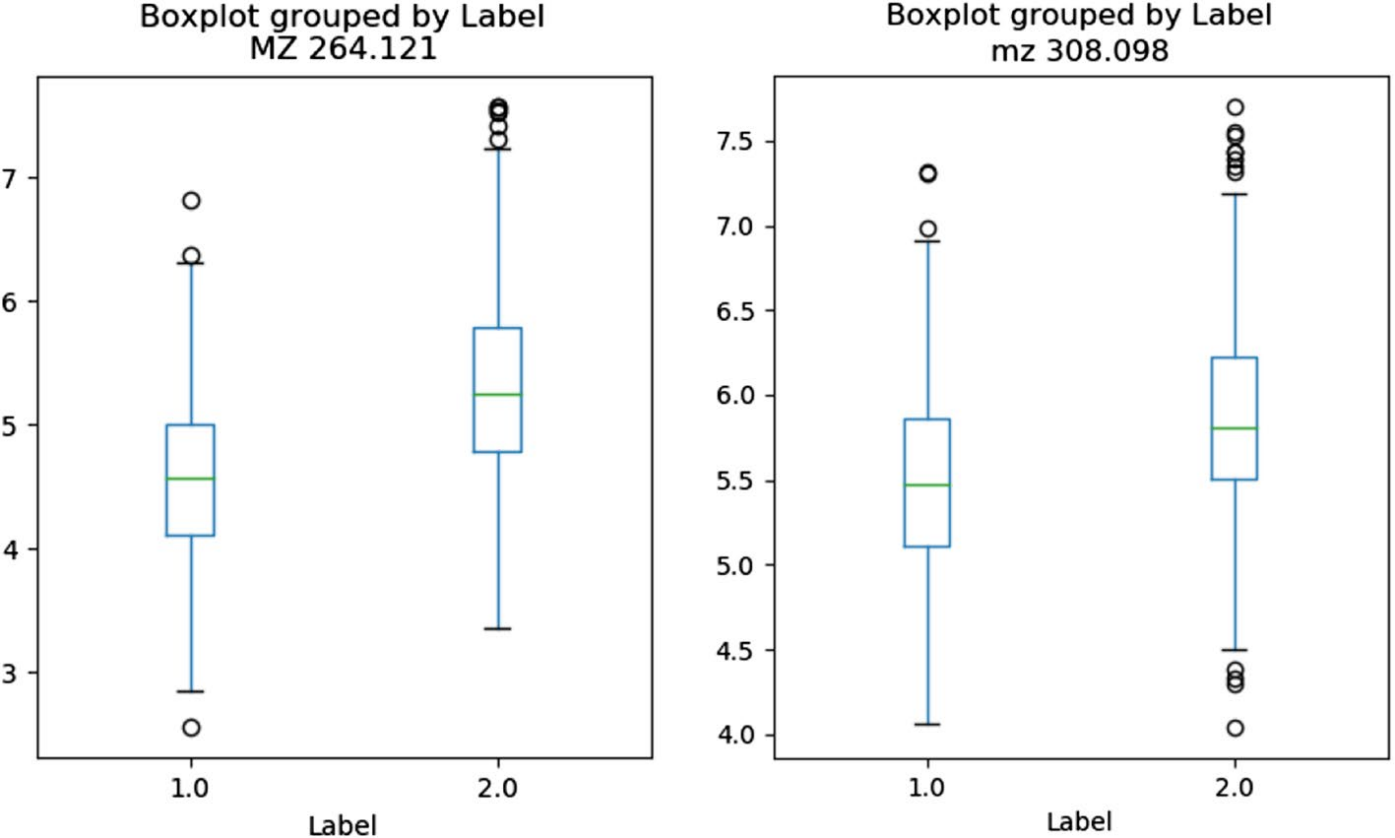
Boxplots concerning relative abundances of features MZ 264.1215224 and MZ 308.0984878 of the LUNG dataset, most likely corresponding to creatine riboside and N-acetylneuraminic acid respectively. Fold changes: 2.57 and 1.43 respectively. Label 1 indicates urine samples of patients without lung cancer. Label 2 indicates urine samples of patients with lung cancer

These features “MZ 264.1215224” and “MZ 308.0984878” most likely correspond to creatine riboside (expected m/z value in the positive mode: 264.1190; mass error: 10 ppm) and N-acetylneuraminic acid (expected m/z value in the negative mode: 308.0987; mass error: 1 ppm), respectively. These two metabolites were described by Mathé et al. [10] as the two most important metabolites to discriminate between lung cancer patients and healthy individuals using Random Forests on metabolomic data from urine samples. Indeed, these two metabolites were significantly higher in the urines of lung cancer patients, as shown in Fig. 2.

### BRAIN

#### Minimally filtered dataset

**Table 4:** BRAIN dataset Accuracy using 3 seeds and 4-fold cross validation: comparison with PLS-DA, Random Forest and best SVM

BRAIN	PD-CR	PD-CR $\ell _2$	PLS-DA	RF (100 trees)	RF (400 trees)	SVM
Accuracy $\%$	92.04	90.9	84.09	88.63	89.39	87.78
AUC	92.08	–	84.33	88.70	89.02	88.53

As shown in Table 4, PD-CR outperformed PD-CR $\ell _2$, PLS-DA, Random Forests (400 trees) and SVM by $1.1 \%$, $7.7 \%$, $2.7 \%$ and $4.3 \%$, respectively for the BRAIN dataset. For this high dimensional dataset, the number of features (25,286) significantly exceeded the number of samples (88) giving a significant drop in the PLS-DA accuracy.

**Fig. 3:**
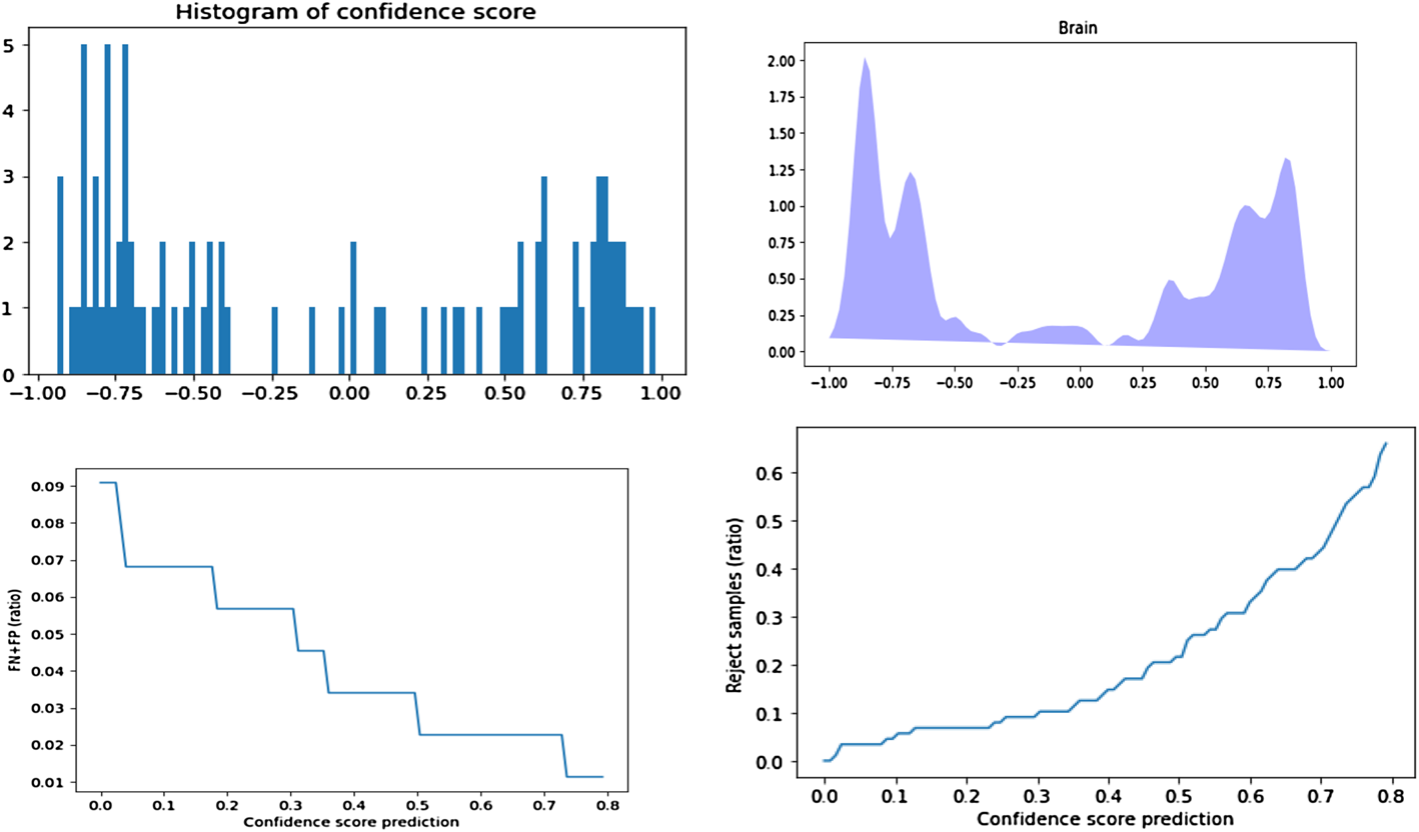
Distribution of the confidence score for the prediction (CSP) on the BRAIN dataset and impact of using CSP for classification with rejection on the false discovery rate (FDR). From left to right and top to bottom: Histogram of the CSP, Kernel density estimation; FDR as a function of CSP after classification with rejection, rate of rejected samples as a function of CSP after classification with rejection. As expected for a pertinent confidence score, the FDR diminishes when using a higher CSP threshold for classification with rejection

Furthermore, as shown in Fig. 3 the accuracy obtained with PD-CR could be further improved by using the CSP to perform classification with rejection. Indeed, most of the samples were classified with a high CSP and if we apply a CSP threshold $\epsilon$ of 0.45, the FDR drops to 0 while only rejecting 10% of the samples. This shows that all the miss-classified samples had a low CSP.

**Table 5:** Top 10 features selected by random forests, PLS-DA, PD-CR and SVM on the BRAIN dataset with 25,286 features

Random forests	PLS-DA	PD-CR	SVM
NEG_MZ147.0867	POS_MZ131.0342	POS_MZ131.0342	POS_MZ131.0342
POS_MZ133.0384	POS_MZ132.0375	POS_MZ132.0375	POS_MZ132.0375
POS_MZ166.0713	POS_MZ166.0713	POS_MZ243.9903	POS_MZ166.0713
POS_MZ228.0182	NEG_MZ147.0288	POS_MZ166.0712	NEG_MZ147.0288
POS_MZ132.5234	NEG_MZ148.0321	NEG_MZ147.0288	NEG_MZ148.0321
POS_MZ173.0306	NEG_MZ149.0329	NEG_MZ148.0321	POS_MZ171.0265
POS_MZ219.0082	POS_MZ171.0265	POS_MZ123.5181	POS_MZ132.0375
NEG_MZ215.0168	POS_MZ132.0375	POS_MZ171.0265	POS_MZ247.9616
POS_MZ171.0265	POS_MZ243.9903	NEG_MZ149.0329	POS_MZ243.9903
POS_MZ319.0510	POS_MZ123.5181	POS_MZ133.0384	NEG_MZ149.0329

As shown in Table 5, most of the top features selected with the 3 methods correspond to different isotopes and adducts of 2-hydroxyglutarate. Indeed, POS_MZ131.0342, POS_MZ132.0375 and POS_MZ133.0384 all correspond to the [M+H-H2O adduct]+ of 2-hydroxyglutarate with C12, and two C13 isotopes respectively. NEG_MZ147.0288, NEG_MZ148.0321 and NEG_MZ149.0329 correspond to the [M-H]- adduct with C12, and two C13 isotopes respectively. POS_MZ166.0713 corresponds to a [M+NH4]+ adduct. POS_MZ171.02645 corresponds to the [M+Na]+ adduct. POS_MZ243.9903 had the same retention time and chromatographic profile as POS_MZ131.0342, suggesting that it was an unknown fragment or adduct of 2-hydroxyglutarate.

**Fig. 4:**
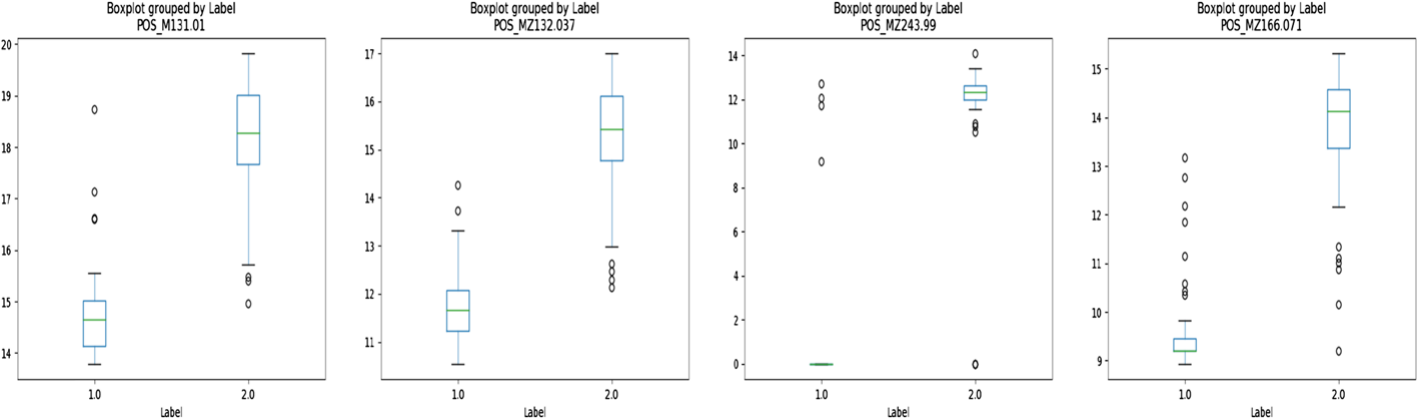
Boxplots concerning relative abundances of features POS_131.0342, POS_132.0375 POS_243.9903 and POS_166.0712 of the BRAIN dataset, most likely corresponding to different adducts of 2-Hydroxyglutarate. Fold changes: 32.9, 35.6, 14.6 and 33.7 respectively. Label 1: samples of tumors with wild type IDH, Label 2: samples of tumors with mutated IDH

2-Hydroxyglutarate is a well-known oncometabolite produced in high quantities by mutated IDH1/2 in gliomas [33]. It is therefore expected that this compound will have a high weight when classifying mutated vs wild-type gliomas as it should be significantly increased in IDH mutated gliomas (as shown in Fig. 4).

Here all four methods selected this important feature among a high dimensional dataset (25,287 features in this case). Adducts and isotopes of 2-hydroxyglutarate with low levels are top selected features using PD-CR indicating that our method is a very sensitive way to identify significant molecules. This result on the minimally filtered dataset also suggest that PC-CR avoids overfiting as no unexpected feature was selected.

#### Comparison to the highly filtered dataset

**Table 6:** Mean accuracy using 4-fold cross validation with 3 different seeds: comparison of methods on the **BRAIN highly filtered ** data set

	PD-CR	PD-CR $\ell _2$	PLS-DA	Random Forests	SVM
Accuracy $\%$	94.31	92.8	93.18	92.04	89.20

As shown in Table 6 the accuracies of the different methods were equivalent and very high when using the highly filtered version of the BRAIN dataset (accuracy being a little lower with SVM).

When PD-CR was used on the highly filtered BRAIN dataset, it lead to similar results as with PD-CR using an $\ell 2$ loss, PLS-DA, Random Forests and SVM. In contrast, it outperformed these methods when using the minimally filtered dataset. In this case, as shown in Table 7 more features were selected. When using the BRAIN dataset for the IDH-mutated vs wild-type classes, most of these additional features were adducts of 2-hydroxyglutarate and are therefore known to be biologically relevant. The additional features that are not adducts of 2-hydroxyglutarate will be investigated in a future study.

**Table 7:** Top 10 features selected by PD-CR in the highly and minimally filtered versions of the **BRAIN** dataset

Identified (495 features)	Large (25,287 features)
POS_M131.0342	POS_MZ131.0342
NEG_M147.02882	POS_MZ132.0375
POS_M85.0291	POS_MZ243.9903
POS_M149.0450	POS_MZ166.0713
NEG_M112.0220	NEG_MZ147.0288
POS_M154.0864	NEG_MZ148.0320
NEG_M171.0847	POS_MZ123.518
NEG_M320.0627	POS_MZ171.0265
POS_M113.0350	NEG_MZ149.0329
POS_M147.1170	POS_MZ133.0384

## Discussion

Machine learning methods are of particular interest for metabolomics studies and are being used increasingly for other omics studies. Herein we introduce a new primal-dual method for supervised classification and feature selection. To our knowledge, a primal-dual method had never been used in this way. We compare this method to three of the most frequently used methods: PLS-DA, Random Forests and SVM, on two metabolomics datasets. Metabolomics datasets tend to be sparse datasets including highly correlated features. PD-CR is particularly suited for this data structure. Hence, for metabolomics, PD-CR appears to be more accurate than the three other methods while selecting biologically relevant features and providing a confidence score for each prediction. An important upside associated with the inclusion of a confidence score for each prediction is that it enables classification with rejection.

We believe that this confidence score is of great value, particularly for applications in medicine. Metabolomics approaches are of particular interest for medical applications. Indeed, they could be used in routine clinical practice as they are relatively inexpensive and can be performed rapidly compared to proteomics, transcriptomics or genomics analyses. More and more studies suggest that metabolomics associated to classification methods are very promising tools for individual personalized medicine [10, 34]. To use metabolomics in routine clinical practice it is paramount to obtain robust, rapid and trustworthy predictions. The confidence score provided with PD-CR adds considerable value to the prediction as it includes a metric that is implicitly used by every physician when they make a medical decision: the probability to make the wrong choice. So far, one of the main obstacles to the use of machine learning in medicine resides in the fact that it is harder to trust the decision of a machine learning method than that of a physician when it comes to health issues. We believe that providing a confidence score associated to the decision would make these new tools more convincing if used in routine clinical practice. Furthermore, this confidence score can be used to perform classification with rejection and reduce the false discovery rate.

Furthermore, this confidence score could be extended to more than 2 classes as follows: We can compute the distances of sample x to all the centroids, respectively. $d_1=\Vert \mu _i-x W\Vert _1$ and we propose a confidence indicator for sample x as follows:

8 $$\begin{aligned} \rho (x) = 1-k \frac{Min(d_1, d_2...,d_k)}{d_1+d_2+...d_k} \end{aligned}$$

Thus, the CSP $\rho (x)$ is a value ranging from 0 to 1. The closer the CSP $\rho (x)$ is to +1 for a predicted class, the higher the confidence will be.

We have shown that PD-CR outperformed the common PLS-DA, Random Forests and SVM methods on both LUNG and BRAIN datasets. We believe that this is partly due to the fact that PD-CR uses a Huber loss. Indeed, the use of the Huber loss with PD-CR leads to a better accuracy than the use of a common $\ell 1$ or $\ell 2$ loss [13]. Note that the l1 loss is not derivable in zero. Moreover the drawback of the term $\Vert Y \mu -XW\Vert _1$ of the l1 loss is that it enforces equality of the two matrices out of a sparse set. Moreover the use of the Huber loss reduces the impact of the presence of outliers in the training set, and therefore leads to a better accuracy than the $\ell 2$ loss, as shown in Tables 2 and 4.

Furthermore we show in Tables 2 and 4 that using PD-CR with an $\ell 2$ loss provides better results than PLSDA which uses the same $\ell 2$ loss. This is probably due to the fact that PLS-DA does not perform feature selection and is known to be prone to overfitting [5].

Moreover, when comparing methods with the minimally filtered and the more filtered versions of the BRAIN dataset, all methods suffered a decrease in accuracy with the minimally filtered dataset (PD-CR keeping the higher accuracy). However the results obtained using the PLS-DA method appeared to be more impacted than those of the Random Forests, SVM and PD-CR. Indeed, the accuracy of PLS-DA significantly decreased when the less filtered dataset was used dropping from 93.18% to 84.09%, compared to a mild decrease in accuracy for the other methods. This can also be explained by the fact that PLS-DA does not perform feature selection and is known to be prone to overfitting [5]. For this reason, several strategies are commonly used to reduce the number of features in metabolomics datasets. Features can be filtered according to the number of detected peaks in all samples, the correct identification of the compound (using the most common adduct) or the presence of isotopes. Working with filtered data has some advantages, including the fact that it appears more biologically relevant to work on less noisy and more reliable data. However, filtering also has some important drawbacks, the most important being the high risk of removing interesting metabolites from the dataset. In the case of the BRAIN dataset, 2-Hydroxyglutarate is a well known metabolite associated to IDH mutation. However, in many metabolomic studies, the goal is to discover potentially unidentified metabolites associated to particular conditions which can only be achieved by including unidentified metabolites. As shown in this work, PD-CR can be applied to both minimally filtered and highly filtered metabolomics datasets.

As it has been previously reported, when designing prediction models, some methods may lead to a more accurate model for a specific dataset while others may be more adapted with other datasets [35]. Indeed, even though we can discuss which machine learning method is the best, most often, researchers try out several machine learning methods on their metabolomics datasets and report the results of the most accurate one. This process has even been automated by some authors [36]. PD-CR is an advanced method, based on recent development in convex optimization and we believe it should be considered by researchers when designing prediction models for metabolomics studies.

Much like the commonly used methods PLS-DA, Random Forests and SVMs, available with [37], our python implementation of PD-CR only requires the tuning of one parameter: $\eta$. This makes the use of PD-CR quite simple, even for non machine learning experts, much like PLS-DA. Note that the tuning of the $\eta$ parameter must be done carefully since it modifies feature selection.

When comparing misclassified patients between methods in an additional analysis, it appeared that in the minimally filtered BRAIN dataset 16/88 tumors were misclassified with at least one method. 2 tumors were misclassified with all methods, 6 with two or three methods and 8 with only one method (3 were misclassfified only with PLS-DA, 4 with Random Forests, 1 with SVM and none with PD-CR). In the LUNG dataset 702/1005 patients were misclassified with at least one method. 68 patients were misclassified with all methods, 240 with two or three methods and 394 with only one method (15 were misclassfified only with PLS-DA, 63 with Random Forests, 305 with SVM and 11 with PD-CR). It therefore appears that PD-CR is the method with the smallest number of false discoveries.

While prior metabolomic studies did not necessarily focus on validating which features the prediction models relied on, it is now admitted that to be trustworthy a model must be based on biologically relevant features and must therefore be interpretable [38]. Indeed, interpretability of machine learning methods [39] is crucial to assess if selected features are biologically relevant. PD-CR offers a straightforward, reliable metric based on the weights of each feature in the model (matrix W).

Conversely, non-linear methods such as Random Forests or non-linear SVM and the linear methods PLS-DA and linear SVM are usually associated to method-specific metrics which makes it difficult to compare features between methods. For Random Forests, the Mean Decrease Impurity (MDI) is usually the default metric for variable importance [40]. It is computed as a mean of the individual trees’ improvement in the splitting criterion produced by each variable. For PLS-DA, the Variable Importance for the Projection (VIP) score is often used. The VIP score is computed by summing the contributions VIN (variable influence) over all model dimensions. For a given PLS dimension *a*, $(VIN)_{ak}^2$ is a function of the squared PLS weight $w_{ak}^2$ [41].

While these metrics offer some insight into the importance of each metabolite in the model these are indirect metrics whereas the weights provided with PD-CR represent the direct quantitative measure of the importance of each feature in the model, very close to regression parameters and can thus directly be used to classify a new sample.

Furthermore, relevant feature selection is necessary for a correct understanding of the biological mechanisms underlying classification. It is well established that when expressed, mutant IDH 1/2 reduces 2-oxo-glutarate to 2-hydroxyglutarate [42]. It was therefore expected for 2-hydroxyglutarate to be a feature of importance as was the case when using PD-CR on the BRAIN dataset for the classification of IDH-mutated vs wild-type gliomas. As the biologically relevant features are known in advance, the BRAIN dataset is a good testing set for this new method. Furthermore, as we described, the features selected with PD-CR in the LUNG dataset are identical to the ones described by Mathé et al. in their original study, which also validates the accurate feature selection performed by PD-CR.

## Conclusion

Herein we propose a recently introduced primal-dual method (PD-CR) for feature selection and classification with rejection. To our knowledge, the primal-dual method has never been used in such fashion. PD-CR includes a sparse regularization factor which is particularly appropriate for high dimensional sparse datasets such as metabolomics datasets.

We highlight the two main results. First, PD-CR is more accurate than PLS-DA, Random Forests and SVM and leads to the selection of biologically relevant features. Second, our method provides a confidence score for each prediction and allows classification with rejection, which can help reduce false discovery rates.

## Supplementary information

**Additional file 1.** Supplementary material: Obtaining metabolomic data for the BRAIN dataset.

## Data Availability

We implemented PD-CR in python. Functions and scripts are freely available at https://github.com/tirolab/PD-CR.
