# Supplementary material for: Primal-dual for classification with rejection (PD-CR): a novel method for classification and feature selection—an application in metabolomics studies
Source: BMC Bioinformatics. 2021 Dec 15;22:594. doi: 10.1186/s12859-021-04478-w (PMC8672607; doi:10.1186/s12859-021-04478-w)
Supplement: Supplementary file 1 — Additional file 1. Supplementary material: Obtaining metabolomic data for the BRAIN dataset. [file 12859_2021_4478_MOESM1_ESM.pdf]

Primal-Dual for Classification with Rejection (PD-CR): A novel method for classification and feature selection. An application in metabolomics studies.

David Chardin, Olivier Humbert, Caroline Bailleux , Fanny Burel-Vandenbos, Valerie Rigau , Thierry Pourcher, Michel Barlaud

December 1, 2021

1 Supplementary material:Obtaining metabolomic data for the BRAIN dataset

1.1 Sample preparation

A fragment of 100mu of each frozen specimen was used for the metabolomic analysis. These fragments were prepared for an unbiased Liquid Chromatography-Mass Spectroscopy (LC-MS) analysis by applying the following procedure :
1. Frozen tissues were placed in microcentrifuge tubes and ground in 1mL of cold methanol (LC-MS grade, Merck Millipore, Molsheim, France) using pestles.
2. Homogenized samples were incubated overnight at -20C then centrifuged at 15000g for 15 minutes.
3. The supernatants were removed and dried using a SpeedVac concentrator (SVC100H, SAVANT, Thermo Fisher Scientific, Villebon-sur-Yvette, France).
4. The lyophilized samples were resuspended in 180μL of 50:50 acetonitrile-H2O mix (LC-MS grade, Merck Millipore) prior to LC-MS/MS analyses.

1.2 LC-MS analysis

Liquid chromatographic analysis was performed using a DIONEX Ultimate 3000 HPLC system (Thermo Fisher Scientific). 10L of each sample was injected onto a Synergi 4m Hydro-RP 80Å, 2503.0mm column (Phenomenex, Le Pecq, France). The mobile phases were composed of : 0.1% formic acid (Thermo Fisher Scientific) in water (A) and 0.1% formic acid in acetonitrile (B). The gradient was set as follows with a flow rate of 0.9mL/min: 0% phase B from 0 to 5min, gradual increase from 0 to 95% of B from 5 to 21min, holding at 95%

of B to 21.5min, rapid decrease from 95 to 0% of B from 21.5 to 22min, holding at 0% B until the end of the analysis at 25min for column equilibration. Mass spectrometry analysis was carried out on a Q Exactive Plus Orbitrap mass spectrometer (Thermo Scientific) with a heated electrospray ionization source, HESI II, operating in both positive and negative mode. High-resolution accurate-mass full-scan MS and top 5 MS2 spectra were collected in a data-dependent fashion at a resolving power of 70 000 and 35 000 at m/z 400, respectively.

1.3 Metabolomic profiling

Metabolomic profiling was performed using MZmine (Version 2.39) [1]. The data obtained from positive and negative ionization modes were analyzed separately. Raw data files were directly imported into MZMine. Mass detection was performed using the Mass detector tool (mass detector : Wavelet transform, MS level 1 ; Noise level 10^4, scale level : 5, Wavelet window size : 30%). Chromatograms were detected using the ADAP chromatogram builder [2] (MS level : 1, Minimum group size in number of scans : 5, Group intensity threshold : $5E2$, minimum highest intensity : 10^4, m/z tolerance : 10 ppm). Peaks were separated using the Peak extender module (M/Z tolerance : 10 ppm, minimum height : 10^4). Retention times were normalized using the retention time calibration module (m/z tolerance : 10 ppm; retention time tolerance (relative) : 10%, minimum height : 10^4). Peaks were then aligned using the RANSAC aligner (random sample consensus) algorithm with a tolerance of 10 ppm in m/z and 1min in retention time. Peaks were then identified using the Human metabolome database (HMDB, version 3.0) with 10 ppm of mass tolerance. Missing values were filled in using the same m/z and RT range gap filler with a tolerance of 10 ppm in m/z. The results obtained with each polarity were combined, and for metabolites that were identified in both modes, we kept the metabolites with higher intensity mean. All metabolites of interest were individually verified (MS and MS2 spectra).

References

[1] Pluskal, T., Castillo, S., Villar-Briones, A., Oresic, M.: MZmine 2: modular framework for processing, visualizing, and analyzing mass spectrometry-based molecular profile data. BMC bioinformatics **11**, 395 (2010). doi:10.1186/1471-2105-11-395

[2] Myers, O.D., Sumner, S.J., Li, S., Barnes, S., Du, X.: One Step Forward for Reducing False Positive and False Negative Compound Identifications from Mass Spectrometry Metabolomics Data: New Algorithms for Constructing Extracted Ion Chromatograms and Detecting Chromatographic Peaks. Analytical Chemistry **89**(17), 8696–8703 (2017). doi:10.1021/acs.analchem.7b00947
